# Redetermination of orotic acid monohydrate

**DOI:** 10.1107/S160053680800562X

**Published:** 2008-03-05

**Authors:** Gustavo Portalone

**Affiliations:** a Chemistry Department, ‘Sapienza’ University of Rome, P. le A. Moro 5, I-00185 Rome, Italy

## Abstract

The crystal structure of the title compound, which is also known as vitamin B_13_ (systematic name: 2,6-dioxo-1,2,3,6-tetra­hydro­pyrimidine-4-carboxylic acid monohydrate), C_5_H_4_N_2_O_4_·H_2_O, was reported for the first time by Takusagawa & Shimada [*Bull. Chem. Soc. Jpn* (1973 ▶), **46**, 2011–2019]. The present redetermination provides more precise values of the mol­ecular geometry. The asymmetric unit comprises a planar diketo tautomer and a solvent water mol­ecule. In the crystal structure, mol­ecules are connected by O—H⋯O, N—H⋯O and C—H⋯O hydrogen bonds involving NH groups, two carbonyl O atoms and the solvent water mol­ecule.

## Related literature

For the previous structure determination, see: Takusagawa & Shimada (1973 ▶). For a general approach to the use of multiple hydrogen-bonding DNA/RNA nucleobases as potential supra­molecular reagents, see: Portalone *et al.* (1999 ▶); Brunetti *et al.* (2000 ▶, 2002 ▶); Portalone & Colapietro (2007 ▶ and references therein). For the computation of ring patterns formed by hydrogen bonds in crystal structures, see: Etter *et al.* (1990 ▶); Bernstein *et al.* (1995 ▶); Motherwell *et al.* (1999 ▶).
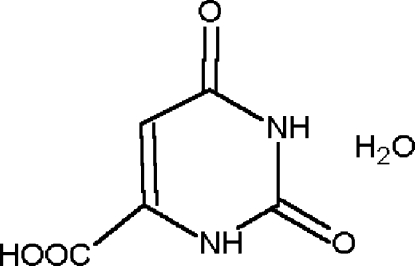


## Experimental

### 

#### Crystal data

C_5_H_4_N_2_O_4_·H_2_O
*M* *_r_* = 174.12
Triclinic, 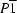

*a* = 5.89854 (14) Å
*b* = 6.92921 (15) Å
*c* = 9.59160 (18) Å
α = 74.6778 (12)°
β = 72.3232 (16)°
γ = 68.447 (2)°
*V* = 342.21 (1) Å^3^
*Z* = 2
Mo *K*α radiation
μ = 0.15 mm^−1^
*T* = 298 (2) K
0.20 × 0.15 × 0.15 mm

#### Data collection

Oxford Diffraction Xcalibur S CCD diffractometer
Absorption correction: multi-scan (*CrysAlis RED*; Oxford Diffraction, 2006 ▶) *T*_min_ = 0.977, *T*_max_ = 0.985
64781 measured reflections
2340 independent reflections
2048 reflections with *I* > 2σ(*I*)
*R*_int_ = 0.017

#### Refinement

*R*[*F* ^2^ > 2σ(*F* ^2^)] = 0.044
*wR*(*F* ^2^) = 0.133
*S* = 1.08
2340 reflections
133 parameters
All H-atom parameters refined
Δρ_max_ = 0.19 e Å^−3^
Δρ_min_ = −0.22 e Å^−3^

### 

Data collection: *CrysAlis CCD* (Oxford Diffraction, 2006 ▶); cell refinement: *CrysAlis RED* (Oxford Diffraction, 2006 ▶); data reduction: *CrysAlis RED*; program(s) used to solve structure: *SIR97* (Altomare *et al.*, 1999 ▶); program(s) used to refine structure: *SHELXL97* (Sheldrick, 2008 ▶); molecular graphics: *ORTEP3* (Farrugia, 1997 ▶); software used to prepare material for publication: *WinGX* (Farrugia, 1999 ▶).

## Supplementary Material

Crystal structure: contains datablocks I, global. DOI: 10.1107/S160053680800562X/kp2160sup1.cif

Structure factors: contains datablocks I. DOI: 10.1107/S160053680800562X/kp2160Isup2.hkl

Additional supplementary materials:  crystallographic information; 3D view; checkCIF report

## Figures and Tables

**Table 1:** Hydrogen-bond geometry (Å, °)

*D*—H⋯*A*	*D*—H	H⋯*A*	*D*⋯*A*	*D*—H⋯*A*
O4—H4⋯O5	0.89 (3)	1.65 (3)	2.5231 (11)	166 (2)
N1—H1⋯O1^i^	0.85 (2)	2.03 (2)	2.8824 (11)	175.2 (19)
N3—H3⋯O2^ii^	0.94 (2)	1.87 (2)	2.8112 (11)	174.6 (18)
O5—H51⋯O2^iii^	0.81 (3)	2.00 (3)	2.7786 (12)	161 (3)
O5—H52⋯O3^iv^	0.82 (3)	1.98 (3)	2.7787 (12)	164 (2)
C5—H5⋯O4^iii^	0.879 (19)	2.740 (19)	3.5922 (13)	163.7 (15)

Symmetry codes: (i) 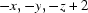; (ii) 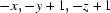; (iii) 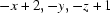; (iv) 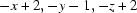.

**Fig. 1.**
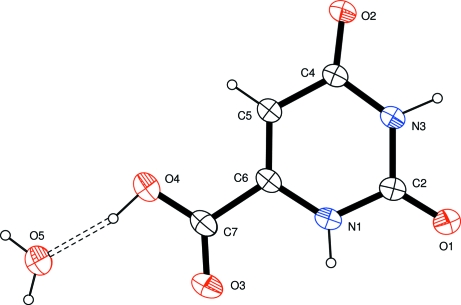
The molecular structure of (I), showing the atom-labelling scheme. Displacements ellipsoids are at the 50% probability level. Hydrogen bonding is indicated by dashed lines.

**Fig. 2.**
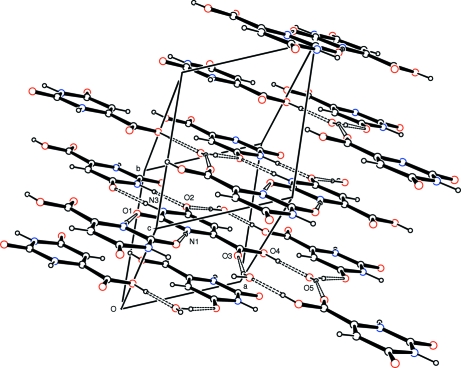
Crystal packing diagram of (I). All atoms are shown as small spheres of arbitrary radii. Hydrogen bonding is indicated by dashed lines.

## supplementary crystallographic information

### Comment

Orotic acid monohydrate, the only effective precursor in the biosynthesis of pyrimidine nucleobases, was determined some 35 years ago (Takusagawa & Shimada, 1973). In this study, 1488 unique reflections were collected at an ambient temperature by photographic techniques using Cu *K*α radiation. 1344 of these, having values significantly above background, were estimated visually and no absorption correction was applied [µ(Cu *K*α) = 15.4 cm^-1^]. The final refinement led to *R* = 0.058 with standard deviations of 0.005Å in C—C bond lengths, As a part of a more general study of multiple hydrogen-bonded DNA/RNA-nucleobases as potential supramolecular reagents (Brunetti *et al.*, 2000, 2002; Portalone *et al.*, 1999; Portalone & Colapietro, 2007), this paper reports a redetermination of the crystal structure of the title compound, (I), with greater precision and accuracy. The asymmetric unit of (I) (Fig. 1) comprises a planar diketo tautomer and a crystal water molecule. The analysis of the crystal packing of (I) shows five N—H···O and O—H···O intermolecular hydrogen bonds (Table 1) which link the molecules into planar layers (Fig. 2). Two kinds of inversion-related N—H···O hydrogen bonds with graph-set motifs *R*^2^_2_(8) (Etter *et al.*, 1990; Bernstein *et al.*, 1995; Motherwell *et al.*, 1999) (Table 1) are formed between NH groups and two carbonyl O atoms (O1^i^ and O2^ii^) [symmetry code: (i) -*x*, -*y*, *z* + 2; (ii) -*x*, -*y* + 1, -*z* + 1] to give a zigzag chain. Each chain is joined by three O—H···O intermolecular hydrogen bonds to form rings of descriptor *R*^4^_4_(12), *R*^4^_3_(13) and *R*^4^_4_(18) through a water molecule (O4—H4···O5, O5—H51···O2^iii^ and O5—H52···O2^iv^; symmetry code: (iii) -*x* + 2, -*y*, -*z* + 1; (iv) -*x* + 2, -*y* - 1, -*z* + 2). The hydrogen-bonded two-dimensional array involves the C5—H5···O4^iii^ intermolecular interaction.

### Experimental

The title compound (0.1 mmol, Sigma Aldrich of 98% purity) was dissolved in water (9 ml) and heated under reflux for 2 h. After cooling the solution to ambient temperature, crystals suitable for single-crystal X-ray diffraction were grown by slow evaporation.

### Refinement

All H atoms were found in a difference map and refined isotropically.

### Crystal data

**Table:**

C_5_H_4_N_2_O_4_·H_2_O	*Z* = 2
*M**_r_* = 174.12	*F*_000_ = 180
Triclinic, *P*1	*D*_x_ = 1.690 Mg m^−^^3^
Hall symbol: -P 1	Mo *K*α radiation λ = 0.71073 Å
*a* = 5.89854 (14) Å	Cell parameters from 64781 reflections
*b* = 6.92921 (15) Å	θ = 3.2–32.0º
*c* = 9.59160 (18) Å	µ = 0.15 mm^−^^1^
α = 74.6778 (12)º	*T* = 298 (2) K
β = 72.3232 (16)º	Plate, colourless
γ = 68.447 (2)º	0.20 × 0.15 × 0.15 mm
*V* = 342.207 (13) Å^3^	

### Data collection

**Table:**

Oxford Diffraction Xcalibur S CCD diffractometer	2340 independent reflections
Radiation source: Enhance (Mo) X-ray source	2048 reflections with *I* > 2σ(*I*)
Monochromator: graphite	*R*_int_ = 0.017
Detector resolution: 16.0696 pixels mm^-1^	θ_max_ = 32.0º
*T* = 298(2) K	θ_min_ = 3.2º
ω and φ scans	*h* = −8→8
Absorption correction: multi-scan(CrysAlis RED; Oxford Diffraction, 2006)	*k* = −10→10
*T*_min_ = 0.977, *T*_max_ = 0.985	*l* = −14→14
64781 measured reflections	

### Refinement

**Table:**

Refinement on *F*^2^	Secondary atom site location: difference Fourier map
Least-squares matrix: full	Hydrogen site location: inferred from neighbouring sites
*R*[*F*^2^ > 2σ(*F*^2^)] = 0.044	All H-atom parameters refined
*wR*(*F*^2^) = 0.133	*w* = 1/[σ^2^(*F*_o_^2^) + (0.0846*P*)^2^ + 0.0376*P*] where *P* = (*F*_o_^2^ + 2*F*_c_^2^)/3
*S* = 1.08	(Δ/σ)_max_ < 0.001
2340 reflections	Δρ_max_ = 0.19 e Å^−^^3^
133 parameters	Δρ_min_ = −0.22 e Å^−^^3^
Primary atom site location: structure-invariant direct methods	Extinction correction: none

### Special details

**Table:**

Experimental. Empirical absorption correction using spherical harmonics, implemented in SCALE3 ABSPACK scaling algorithm.
Geometry. All e.s.d.'s (except the e.s.d. in the dihedral angle between two l.s. planes) are estimated using the full covariance matrix. The cell e.s.d.'s are taken into account individually in the estimation of e.s.d.'s in distances, angles and torsion angles; correlations between e.s.d.'s in cell parameters are only used when they are defined by crystal symmetry. An approximate (isotropic) treatment of cell e.s.d.'s is used for estimating e.s.d.'s involving l.s. planes.
Refinement. Refinement of *F*^2^ against ALL reflections. The weighted *R*-factor *wR* and goodness of fit *S* are based on *F*^2^, conventional *R*-factors *R* are based on *F*, with *F* set to zero for negative *F*^2^. The threshold expression of *F*^2^ > σ(*F*^2^) is used only for calculating *R*-factors(gt) *etc*. and is not relevant to the choice of reflections for refinement. *R*-factors based on *F*^2^ are statistically about twice as large as those based on *F*, and *R*- factors based on ALL data will be even larger.

### Fractional atomic coordinates and isotropic or equivalent isotropic displacement parameters (Å^2^)

**Table:**

	*x*	*y*	*z*	*U*_iso_*/*U*_eq_	
O1	−0.14403 (15)	0.19593 (14)	0.87497 (9)	0.0437 (2)	
O2	0.33017 (14)	0.40869 (13)	0.43298 (8)	0.0402 (2)	
O3	0.67729 (18)	−0.25757 (17)	0.92057 (11)	0.0575 (3)	
O4	0.94015 (16)	−0.17721 (14)	0.70790 (10)	0.0430 (2)	
H4	1.045 (5)	−0.276 (4)	0.759 (3)	0.072 (6)*	
N1	0.27952 (15)	0.03091 (13)	0.83018 (9)	0.03108 (19)	
H1	0.248 (4)	−0.038 (3)	0.918 (2)	0.052 (4)*	
C2	0.06502 (18)	0.17428 (15)	0.79307 (10)	0.0302 (2)	
N3	0.09988 (15)	0.29328 (13)	0.65310 (8)	0.03059 (19)	
H3	−0.050 (4)	0.391 (3)	0.630 (2)	0.054 (5)*	
C4	0.32531 (17)	0.28849 (14)	0.55477 (10)	0.0284 (2)	
C5	0.54286 (17)	0.13666 (14)	0.60218 (10)	0.0295 (2)	
H5	0.689 (3)	0.127 (3)	0.540 (2)	0.042 (4)*	
C6	0.51057 (17)	0.01317 (13)	0.73716 (9)	0.02706 (19)	
C7	0.72006 (19)	−0.15575 (15)	0.79814 (11)	0.0325 (2)	
O5	1.28887 (17)	−0.45822 (16)	0.81521 (11)	0.0523 (3)	
H51	1.421 (5)	−0.460 (4)	0.757 (3)	0.080 (7)*	
H52	1.313 (5)	−0.528 (4)	0.896 (3)	0.082 (7)*	

### Atomic displacement parameters (Å^2^)

**Table:**

	*U*^11^	*U*^22^	*U*^33^	*U*^12^	*U*^13^	*U*^23^
O1	0.0285 (4)	0.0499 (5)	0.0329 (4)	−0.0054 (3)	−0.0022 (3)	0.0094 (3)
O2	0.0291 (4)	0.0447 (4)	0.0298 (4)	−0.0065 (3)	−0.0079 (3)	0.0154 (3)
O3	0.0390 (5)	0.0612 (6)	0.0449 (5)	−0.0062 (4)	−0.0138 (4)	0.0266 (4)
O4	0.0302 (4)	0.0449 (4)	0.0385 (4)	−0.0003 (3)	−0.0112 (3)	0.0054 (3)
N1	0.0289 (4)	0.0311 (4)	0.0247 (3)	−0.0063 (3)	−0.0080 (3)	0.0067 (3)
C2	0.0277 (4)	0.0308 (4)	0.0252 (4)	−0.0071 (3)	−0.0065 (3)	0.0037 (3)
N3	0.0245 (4)	0.0319 (4)	0.0258 (3)	−0.0047 (3)	−0.0077 (3)	0.0070 (3)
C4	0.0260 (4)	0.0289 (4)	0.0244 (4)	−0.0064 (3)	−0.0078 (3)	0.0041 (3)
C5	0.0243 (4)	0.0308 (4)	0.0258 (4)	−0.0045 (3)	−0.0072 (3)	0.0029 (3)
C6	0.0268 (4)	0.0251 (4)	0.0257 (4)	−0.0045 (3)	−0.0106 (3)	0.0014 (3)
C7	0.0306 (4)	0.0297 (4)	0.0322 (4)	−0.0049 (3)	−0.0137 (3)	0.0038 (3)
O5	0.0287 (4)	0.0598 (6)	0.0426 (5)	0.0010 (4)	−0.0082 (3)	0.0118 (4)

### Geometric parameters (Å, °)

**Table:**

O1—C2	1.2241 (12)	N3—C4	1.3716 (12)
O2—C4	1.2418 (10)	N3—H3	0.94 (2)
O3—C7	1.2056 (13)	C4—C5	1.4387 (12)
O4—C7	1.3040 (13)	C5—C6	1.3525 (12)
O4—H4	0.89 (3)	C5—H5	0.879 (19)
N1—C6	1.3656 (12)	C6—C7	1.5012 (12)
N1—C2	1.3702 (12)	O5—H51	0.81 (3)
N1—H1	0.85 (2)	O5—H52	0.82 (3)
C2—N3	1.3772 (11)		
			
C7—O4—H4	104.3 (16)	N3—C4—C5	115.74 (8)
C6—N1—C2	122.36 (8)	C6—C5—C4	118.56 (8)
C6—N1—H1	126.4 (14)	C6—C5—H5	124.1 (11)
C2—N1—H1	111.2 (14)	C4—C5—H5	117.3 (11)
O1—C2—N1	123.81 (8)	C5—C6—N1	122.13 (8)
O1—C2—N3	121.35 (8)	C5—C6—C7	124.03 (9)
N1—C2—N3	114.83 (8)	N1—C6—C7	113.84 (8)
C4—N3—C2	126.29 (7)	O3—C7—O4	125.70 (9)
C4—N3—H3	120.3 (12)	O3—C7—C6	120.28 (10)
C2—N3—H3	113.3 (12)	O4—C7—C6	114.02 (8)
O2—C4—N3	119.61 (8)	H51—O5—H52	110 (2)
O2—C4—C5	124.64 (9)		
			
C6—N1—C2—O1	−178.86 (10)	C4—C5—C6—N1	−1.20 (15)
C6—N1—C2—N3	2.20 (15)	C4—C5—C6—C7	178.02 (9)
O1—C2—N3—C4	177.12 (10)	C2—N1—C6—C5	0.18 (16)
N1—C2—N3—C4	−3.91 (15)	C2—N1—C6—C7	−179.11 (9)
C2—N3—C4—O2	−178.17 (10)	C5—C6—C7—O3	178.85 (12)
C2—N3—C4—C5	2.97 (15)	N1—C6—C7—O3	−1.87 (15)
O2—C4—C5—C6	−179.05 (10)	C5—C6—C7—O4	−1.13 (15)
N3—C4—C5—C6	−0.26 (14)	N1—C6—C7—O4	178.15 (9)

### Hydrogen-bond geometry (Å, °)

**Table:**

*D*—H···*A*	*D*—H	H···*A*	*D*···*A*	*D*—H···*A*
O4—H4···O5	0.89 (3)	1.65 (3)	2.5231 (11)	166 (2)
N1—H1···O1^i^	0.85 (2)	2.03 (2)	2.8824 (11)	175.2 (19)
N3—H3···O2^ii^	0.94 (2)	1.87 (2)	2.8112 (11)	174.6 (18)
O5—H51···O2^iii^	0.81 (3)	2.00 (3)	2.7786 (12)	161 (3)
O5—H52···O3^iv^	0.82 (3)	1.98 (3)	2.7787 (12)	164 (2)
C5—H5···O4^iii^	0.879 (19)	2.740 (19)	3.5922 (13)	163.7 (15)

Symmetry codes: (i) −*x*, −*y*, −*z*+2; (ii) −*x*, −*y*+1, −*z*+1; (iii) −*x*+2, −*y*, −*z*+1; (iv) −*x*+2, −*y*−1, −*z*+2.
